# FTY720 Attenuates Angiotensin II-Induced Podocyte Damage via Inhibiting Inflammatory Cytokines

**DOI:** 10.1155/2017/3701385

**Published:** 2017-02-07

**Authors:** Ke Su, Ping Zeng, Wei Liang, Zhengyu Luo, Yiman Wang, Xifeng Lv, Qi Han, Miao Yan, Cheng Chen

**Affiliations:** ^1^Department of Nephrology, Renmin Hospital of Wuhan University, 238 Jiefang Road, Wuhan 430060, China; ^2^First School of Clinical Medicine, Renmin Hospital of Wuhan University, 238 Jiefang Road, Wuhan 430060, China

## Abstract

FTY720, a new chemical substance derived from the ascomycete* Isaria sinclairii*, is used for treating multiple sclerosis, renal cancer, and asthma. Sphingosine 1-phosphate (S1P) is a bioactive sphingolipid metabolite and exists in red blood cells. FTY720 is a synthetic S1P analog which can block S1P evoking physiological effects. Recently studies show that S1P was participating in activated inflammation cells induced renal injury. The objective of this study was to assess the protective effect of FTY720 on kidney damage and the potential mechanism of FTY720 which alleviate podocyte injury in chronic kidney disease. In this study, we selected 40 patients with IgA nephropathy and examined their clinical characteristics. Ang II-infusion rat renal injury model was established to evaluate the glomeruli and tubulointerstitial lesion. The result showed that the concentration of S1P in serum and urine was positively correlated with IgA nephropathy patients' renal injury. FTY720 could reduce renal histological lesions induced by Ang II-infusion in rats. Moreover, FTY720 decreased S1P synthesis in Ang II-infusion rats via downregulation of inflammatory cytokines including TNF-*α* and IL-6. In addition, FTY720 alleviated exogenous S1P-induced podocyte damage. In conclusion, FTY720 is able to attenuate S1P-induced podocyte damage via reducing inflammatory cytokines.

## 1. Introduction

Chronic kidney disease (CKD) includes a broad variety of pathologies, especially the chronic impairment of renal excretory function primarily arising from injury to renal structures. The majority forms of CKD are irretrievable and progressive. Renal damage comprises nephron loss, fibrosis, and renal vasculature changes [1]. CKD results from various causes including inflammatory and infiltrative diseases, nephritis, diabetes, hypertension, and renal and systemic infections. Renal inflammation, featured by the infiltration of inflammatory cells to kidney parenchyma, is a main pathologic process of various CKD [2–4]. Inflammatory infiltration results in the initiation and development of CKD. Inflammatory cell infiltration in the interstitium and persistent fibrogenesis involves several pathways, for example, stimulation of tubular chemokine expression, inflammatory cytokines, various growth factors, and monocyte chemotactic proteins [5]. Proteinuria represents a robust marker for progression of CKD [5]. The integrity of the glomerular filtration barrier relies on its three-layered structure such as endothelium, glomerular basement membrane (GBM), and podocytes. Augmented intraglomerular hydraulic pressure or glomerular filtration barrier impairment might cause glomerular/overload proteinuria [5]. Hematuria and proteinuria are the primary early-stage clinical features of CKD and can provoke proinflammatory and/or profibrotic effects, directly eliciting chronic tubulointerstitial damage.

Sphingosine 1-phosphate (S1P) is a bioactive sphingolipid metabolite that acts both as an extracellular mediator and as intracellular second messenger. S1P signaling mediates the pathogenesis of various diseases, such as inflammatory diseases, osteoporosis, and arthritis [6–8]. A number of cell types such as red blood cells (RBC), platelets, endothelial cells, and neutrophils secrete S1P. RBC are the main source of S1P in plasma [9, 10]. Sphingosine kinases (SPHK) including two isoforms, SPHK1 and SPHK2 [11], are crucial regulators of S1P. SPHK1 is predominantly localized to the cytosol and translocates to the plasma membrane upon activation in eukaryotic cells. SPHK2 is found primarily in the nucleus [11]. S1P, via elevating its intracellular content through sphingolipid metabolism and binding to its receptors, controls a number of physiological/pathological processes, for example, cell proliferation, autophagy, migration, and angiogenesis. These processes are associated with tumor growth, metastasis, and invasion. S1P may interact with a family of G protein-coupled receptors (S1PRs), also known as endothelial differentiation gene (EDG) receptors [12], which affects the cellular responses to S1P. S1PRs, including S1P receptors 1 to 5 (S1PR1–S1PR5), are expressed in different tissue cells specifically [13, 14]. S1PR1 is extensively expressed in brain, heart, lung, liver, and spleen and, to a lesser extent, in thymus, kidney, renal medulla, glomeruli, and muscle [15–20]. Particularly, S1PR1 plays a critical role in the development of vascular lesions, progression of atherosclerosis, cancer, autoimmune disease, or multiple sclerosis [21–23]. Furthermore, deletion of S1PR1 intensifies kidney damage and inflammation [24] and S1PRs activation in kidneys and bone marrow-derived cells decreases inflammation [25, 26]. Overall, it is widely speculated that S1PR1 is implicated in regulating vascular tone and participating in renal damage under pathologic conditions. However, little is known about the role of S1PR1 in the development of renal damage.

FTY720, a synthetic S1P analog, is phosphorylated by SPHK1 and SPHK2 into its bioactive form, FTY720-phosphate [27–29]. FTY720-phosphate functions as a noncompetitive inhibitor of various S1PRs [30, 31], such as S1PR1, S1PR3, S1PR4, and S1PR5, but not S1PR2, receptors [27, 32, 33]. FTY720-phosphate hinders S1P signaling through prompting the internalization and subsequent degradation of S1PRs [30]. Particularly, FTY720 has shown an extraordinary protective effect against autoimmune myocarditis [34], multiple sclerosis [27, 35], uveoretinitis [36], and atherosclerosis [37]. Clinical trials have been conducted to test its preventive effect on the rejection of renal transplant [38, 39]. Moreover, FTY720 administration relieved ovariectomy-induced osteoporosis [40] and mitigated lipopolysaccharide-induced arthritis in mice [41]. In this study, we used FTY720 to block S1P-elicited physiological effects. While previous studies suggested that FTY720 repressed immune response [38, 40], the mechanism(s) by which FTY720 modulates inflammatory diseases are poorly understood. In this study, we investigated the mechanisms behind FTY720 inhibition of the inflammatory response and alleviation of podocyte injury in chronic kidney disease.

## 2. Materials and Methods

### 2.1. Human Studies

Peripheral blood was collected from IgA nephropathy patient and healthy subjects. All patients were subjected to renal biopsy and the histological diagnoses of IgA nephropathy in Renmin Hospital of Wuhan University within 2014~2015. The histological grading of patients with IgA nephropathy was classified by Lee's grades I~IV. Patients who have systemic disease of SLE, Henoch-Schönlein Purpura Nephritis, and diabetic and chronic liver diseases were clinically excluded. Patients who received steroid therapy and immune depressant treatment were excluded. We collected patients' clinical information including age, sex, presence of hypertension (blood pressure > 140/95 mmHg or requirement for antihypertensive therapy), plasma creatinine, blood urea nitrogen, high-density lipoprotein, triglyceride, complement (C3 and C4), serum albumin, serum total protein, and 24 h urinary protein. The 24-hour urine protein excretion was tested by sulfosalicylic acid method. The renal biopsy tissues from patients with IgA nephropathy were stored immediately at −80°C for further tissue freezing section of pathological analysis. Renal tissue adjacent to carcinoma was collected as a normal control. These studies were approved by the hospital's Institutional Ethics Committee (Renmin Hospital of Wuhan University, China), and written informed consent was obtained from all patients.

### 2.2. ELISA

Plasma and urine S1P concentration in IgA nephropathy patient, healthy person, and rats were assessed by S1P ELISA (Echelon Biosciences, Salt Lake City, UT). Blood was allowed to clot and was then centrifuged and aliquots of serum were stored at −70°C before S1P analyses. The 24 h urine was collected for S1P level analyses. Plasma and urine were applied to the ELISA plate at 30 *μ*g protein/well. ELISA was performed according to manufacturer's instructions. Results were confirmed by comparison to S1P concentration determined by microplate reader (BioTek, VT, USA) at OD 450 nm. Concentration of S1P was calculated in the samples by comparing the OD of the samples to the standard curve.

### 2.3. Animal

Animal experiments were performed according to the Guide for the Care and Use of Laboratory Animals of Wuhan University. Thirty-six male SPF Wistar rats weighing 160 ± 10 g were purchased from SJA experimental animals company (Hunan, China) and were maintained at a controlled temperature (23 ± 2°C) and humidity (55 ± 5%) with a light/dark cycle of 12/12 h and ad libitum fed with rodent chow and water. The rats were embedded with osmotic mini-pump (Alzet model 2002 or 2004, CA), mini-pump subcutaneous implant method as described in our previous studies [42, 43]. Rats were randomly subjected to normal saline infusion, or subcutaneous continuous Ang II-infusion at 400 ng/kg·min concentration, or Ang II at 400 ng/kg·min + FTY720 0.5 g/kg·d by means of intragastric administration for 14 or 28 days. 24-hour urine was collected in metabolic cages and urinary albumin excretion rate was measured at days 14 and 28. At the end of 14- or 28-day treatment, rats were sacrificed. Blood samples were immediately collected in EDTA-containing tubes and serum-separating tubes to separate the plasma and serum. Serum Ang II concentration, S1P concentration, kidney function blood urea nitrogen (BUN) and creatinine, and serum albumin level were measured. 24-hour urine protein excretion was tested by sulfosalicylic acid method. The kidneys were dissected and rinsed with cold isotonic saline and weighed. The right kidney tissues were fixed in 10% neutralized formalin for histological analysis.

### 2.4. Angiotensin II Radioimmunoassay

Rat plasma and kidney angiotensin II radioimmunoassay was performed in 12 × 75 mm polypropylene culture tubes as described in the Amersham RIA procedure (Amersham Corporation, Arlington Heights, IL). Radioactivity was determined in a T Tri-Carb 4810TR Liquid Scintillation Analyzer (PerkinElmer, Boston, USA).

### 2.5. Hematoxylin-Eosin Staining

Sections of rat kidney were stained with hematoxylin for 10 minutes. Subsequently, they were washed under running tap water for 5 minutes, dried on a hot plate, and stained with 0.5% eosin in 96% ethanol for 5 minutes. The sections were rapidly rinsed in 95% ethanol and dehydrated in 2 changes of absolute ethanol for 5 minutes each. Slides were dehydrated, cleared in xylene, and mounted in resinous medium.

### 2.6. Immunohistochemical Staining

The sections were washed with PBS (pH 7.5) and incubated in protein blocking solution (0.5% normal goat serum in PBS, v/v) for 30 min. The sections were then incubated with antibody against rat TNF-*α* (1 : 100, GTX110520, Novus, Saint Charles, MI, USA) and rat IL-6 (1 : 100, Novus, Saint Charles, MI, USA) in a humidified chamber for 4°C overnight [44], rinsed with PBS 3 times, and incubated with peroxidase-conjugated secondary antibody (1 : 100, DAKO, Glostrup, Denmark) for 1 h at room temperature. To detect positive reactions, the slides were incubated with stable 3,39-diaminobenzidine (DAB) for 5–10 min (Zhongshan Jinqiao Biotechnology, Beijing, China). The sections were rinsed with distilled water, counterstained with Gill's hematoxylin for 1 min (Sigma, St. Louis, MO), and observed under a BX53 Olympus microscope.

To quantify glomerular and tubulointerstitial lesion, immunoperoxidase kidney sections were examined under ×20 magnification, using an Olympus BX40 microscope (Olympus Optical, London, UK) mounted with a Photonic Science Color Coolview digital camera (Photonic Science, East Sussex, UK). Digital images were captured and analyzed using Image-Pro Plus software (Media Cybernetics, Silver Spring, MD, USA), and color segmentation was performed for each slide individually, defining pixels that contained appropriate coloration. For each slide, 20 consecutive glomeruli or cortical areas were defined as an “area of interest,” and the percentage of each cross-sectional area stained with the defined color was calculated. The final value for each slide was derived as the mean percentage area stained from 20 areas.

### 2.7. Cell Culture and Treatment

A conditionally immortalized murine podocytes were kindly provided by Dr. Peter Mundel (Mount Sinai School of Medicine, New York). Podocytes were maintained in RPMI 1640 medium (HyClone, USA) containing 10% heat-inactivated fetal calf serum (Gibco, USA), 100 U/mL penicillin G, and 100 *μ*g/mL streptomycin in an incubator with 5% CO_2_. During podocyte proliferation, the medium was mixed with 10 U/mL recombinant mouse interferon-*γ* (Sigma, USA), and the cells were maintained at 33°C. Then podocytes were cultured at 37°C to induce differentiation without interferon-*γ* for 10–14 days. 15–25 passages of podocytes were used in the following experiments.

Cells were cultured in serum-free RPMI 1640 for at least 8 h and pretreated with 5 *μ*mol/L FTY720 (Cayman Chemicals, Ann Arbor, MI, USA) for 30 min followed by treatment with 2 *μ*mol/L S1P (Sigma Chemical Co.) for 24 h.

### 2.8. Western Blot

Western blot was executed as previously described [45]. Briefly, 30 *μ*g protein lysates were resuspended in a reduced sample buffer and electrophoresed on a Tris gel, blotted to NC membrane, and subsequently probed with primary antibodies including FAK (1 : 500; Abcam, Cambridge, MA, USA), rabbit polyclonal to SPHK1 (1 : 500; Abcam, Cambridge, MA, USA), Alexa Fluor 680 (Invitrogen), and IRDye 800 (LI-COR Biosciences). A horseradish peroxidase-conjugated goat anti-rabbit antibody was then added. The signal was detected through autoradiography using enhanced chemiluminescence (ECL Plus, General Electric Healthcare, Milwaukee, WI) with the Odyssey infrared imaging system (LI-COR Biosciences) and quantification was performed using Odyssey software. Western blot data were evaluated as follows: the gray value of the western blot bandings was examined by Image-Pro Plus (Media Cybernetics, Inc.) in the control and experimental groups; the gray value of interest protein of each group was divided by the gray value of internal reference protein itself.

### 2.9. Real-Time PCR

Total RNA was extracted with Trizol (Invitrogen). Total RNA (2 mg) was used for first-strand cDNA synthesis with RevertAid™ First-Strand cDNA Synthesis Kit (Fermentas, Vilnius, LTU). PCR was performed in the presence of SYBR green using a 7500 Fast Real-Time PCR System. All PCR reactions were run in triplicate and repeated at least three times. Differences were calculated according to the relative quantization method using the *β*-actin gene to calibrate. The primers for rat IL-6 forward: 5′-TGATGGATGCTTCCAAACTG-3′, reverse: 5′-GAGCATTGGAAGTTGGGGTA-3′; rat TNF-*α* forward: 5′-ACTGAACTTCGGGGTGATTG-3′, reverse: 5′- GCTTGGTGGTTTGCTACGAC-3′.

### 2.10. Immunofluorescence

Conditionally immortalized murine podocytes were cultured on coverslips in a 24-well plate. Cells were fixed with 4% paraformaldehyde for 30 min at room temperature, washed, and permeabilized with 0.5% Triton X-100 for 5 min. The cells were incubated with phalloidin-Alexa 488 (1 : 40, Invitrogen, Carlsbad, CA) 4°C for 20 min. ProLong Gold Antifade reagent along with DAPI (Invitrogen) was used to mount the coverslips to slides. The cells were visualized using a confocal for fluorescence to detect the subcellular distribution of the actin cytoskeleton. The green fluorescence represents F-actin; and the blue fluorescence represents the cell nucleus.

### 2.11. Electron Microscopy

Renal cortex (1 mm^3^) from each rat was cut into small pieces and fixed in 2.5% glutaraldehyde in 0.1 mol/L phosphate buffer (pH 7.4) at 4°C for several days. After washing in phosphate buffer and postfixing in 1% OsO_4_ for 2 h, the fixed material was dehydrated and embedded in Epon 812 (Okenshoji, Tokyo, Japan). Ultrathin sections were prepared and stained with uranyl acetate and lead citrate and examined with a Hitachi H7100 electron microscope (Hitachi, Yokohama, Japan).

### 2.12. Wound-Healing Assay

Wound-healing assay was executed as previously described [46]. Cells were plated at a density of 1 × 10^6^ cells/mL in a 6-well plate and grown to 100% confluence. The cells were serum starved for 12 h. A wound was scratched at the inner bottom of each well using a P-200 pipette tip. The cells were washed twice with phosphate buffer saline (PBS) to remove the cell debris; then serum-free RPMI 1640 medium containing 10 *μ*g/mL mitomycin C and irritant was added to each well. Cells within the same field were photographed at 0 and 24 h after the cells were scratched. The percentage of wound closure was calculated using the following formula, in which S is the surface area of the wound field.

### 2.13. Cell Adhesion Assay

Cell adhesion assay was executed as previously described [46]. The 96-well plates were coated with 50 *μ*L fibronectin (Fn) (20 *μ*g/mL) or Matrigel (200 *μ*g/mL) (Becton Dickinson, Heidelberg, Germany) at 37°C for 2 h, then washed twice with PBS, and blocked with serum-free DMEM + 2% BSA for 30 min at 37°C. The cells were treated with irritant for 24 h at 37°C in a humidified incubator supplemented with 5% carbon dioxide. The treated podocyte cells were harvested with 0.25% trypsin-EDTA and resuspended to a density of 1 × 10^5^ cells/mL in serum-free RPMI 1640 medium and then seeded at a density of 100 *μ*L per well in the previously coated 96-well plates. Then, the cells were allowed to attach to the plates for 1.5 h at 37°C.

After the cells were washed gently with PBS twice, 20 *μ*L of 3-(4,5-dimethyl-2-thiazolyl)-2,5-diphenyltetrazolium bromide (MTT) was added to each well for 4 h. Then, the MTT was removed, and 200 *μ*L of dimethylsulfoxide (DMSO) was added to each well. The optical density (OD) at 570 nm of each well was measured with a microplate reader 15 min later. The experiments were performed three times. The control cells were not treated with matrine. The cell adhesion ratio was calculated using the following formula:

(1) $$\begin{array}{c} \text{The cell adhesion ratio}=\frac{OD570_{\text{treated group}}}{OD570_{\text{untreated group}}}\times 100\%. \end{array}$$

### 2.14. Statistical Analyses

Data are reported as mean ± standard error. Data were analyzed using Student's two-tailed unpaired or paired (fed/fasting experiments) *t*-tests. Statistical analysis of data having equal variance was performed by one-way or two-way analysis of variance (ANOVA) followed by Tukey's post hoc test when appropriate. Associations between variables were investigated using Spearman correlation coefficient with SPSS 13.0 software. The differences at *P* < 0.05 were considered statistically significant. The data were analyzed using SPSS 13.0.

## 3. Results

### 3.1. The Concentration of S1P in Serum and Urine Was Positively Correlated with Chronic Kidney Disease Renal Injury

Mild and moderate clinical IgA nephropathy are characterized by hematuria and proteinuria. Thus, we selected IgA nephropathy as the representative disease of chronic kidney disease. Lee's histological classification (grades I~IV) was employed. Total 40 patients, average age 36.80 ± 13.77 years (range within 19–67), have varying degrees of hematuria and proteinuria, with or without renal dysfunction. We randomly selected 10 healthy volunteers and collected their serum and urine. The clinical characteristics of all participants are presented in Table 1. In this study, of 40 IgA nephropathy patients 18 were male. There was a slight female preponderance. The 24 h urinary protein excretion was 0.56–4.3 g/24 h. The patients' gender, age, serum urea nitrogen, serum creatinine, HDL, complement C3, and systolic and diastolic blood pressure did not show statistically significant differences (*P* > 0.05). However, the serum total protein, albumin, 24 h urinary protein excretion, complement C4, serum IgA, cholesterol, and triglycerides exhibited statistically significant differences (*P* < 0.05). In IgA nephropathy group, serum total protein and albumin levels were lower than those of the control group, but 24 h urine protein, complement C4, serum IgA, cholesterol, and triglycerides in IgA nephropathy group were significantly increased versus the control group (data not shown).

**Figure 1:**
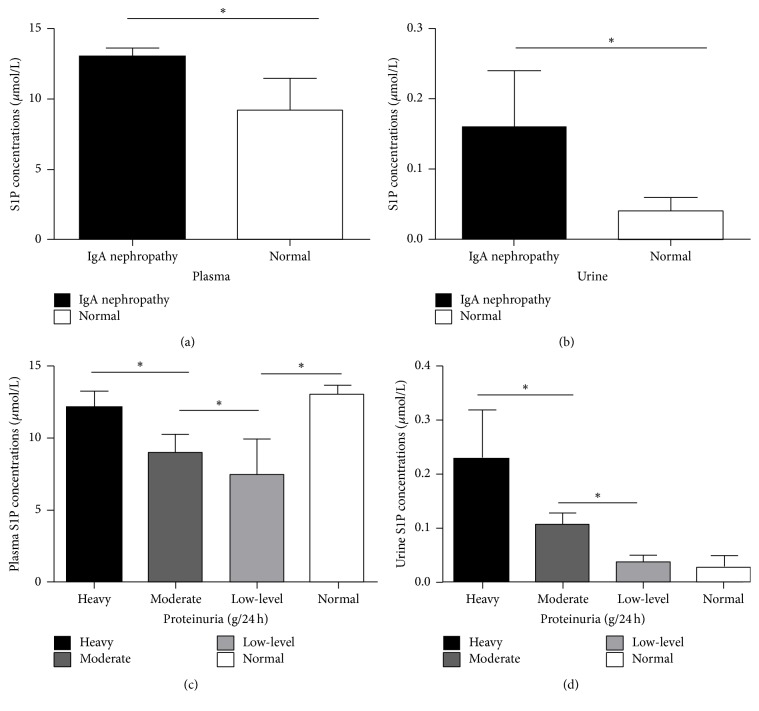
The concentration of S1P in serum and urine was positively correlated with chronic kidney disease renal injury. Forty IgA nephropathy patients and 10 healthy volunteers were selected. The 24 h urine and plasma were collected. Concentrations of S1P in plasma and urine were measured by using ELISA. (a) Concentrations of S1P in plasma in IgA nephropathy patients and healthy volunteers. (b) Concentrations of S1P in 24 h urine in IgA nephropathy patients and healthy volunteers. (c) Concentrations of S1P in plasma in IgA nephropathy patients with different level of proteinuria and healthy volunteers. (d) Concentrations of S1P in 24 h urine from IgA nephropathy patients with different level of proteinuria and healthy volunteers. Data were expressed as means ± standard error of the mean from the independent sample in the same group. ^*∗*^*P* < 0.05, versus normal group in (a) and (b). ^*∗*^*P* < 0.05, versus heavy group or low-level group in (c) and (d).

Concentrations of S1P in plasma and urine were measured using ELISA. As shown in Figures 1(a) and 1(b), there was a significant difference between the control and IgA nephropathy groups in plasma (9.22 ± 2.2 versus 13.00 ± 0.60 *μ*mol/L, *P* < 0.05) and urine concentrations of S1P (0.04 ± 0.02 versus 0.16 ± 0.08 *μ*mol/L, *P* < 0.05). The concentration of S1P in plasma and urine in IgA nephropathy patients was significantly higher than healthy volunteers.

Based on the total amount of 24 h urinary protein excretion, IgA nephropathy patients were divided into three groups: low-level proteinuria (0.15~1 g/24 h, 13 cases), moderate proteinuria (1~3.5 g/24 h, 16 cases), and heavy proteinuria (≥3.5 g/24 h, 11 cases). The plasma S1P levels in heavy proteinuria, moderate proteinuria, and low-level proteinuria were 12.15 ± 1.05, 8.98 ± 1.23, and 7.51 ± 2.38 *μ*mol/L, respectively. Plasma S1P levels showed statistically significant differences between these three groups (*P* < 0.05) (Figure 1(c)). In addition, the urine S1P levels in heavy proteinuria, moderate proteinuria, and low-level proteinuria were 0.23 ± 0.09, 0.11 ± 0.02, and 0.04 ± 0.01 *μ*mol/L, respectively, which showed statistically significant differences (*P* < 0.05) (Figure 1(d)). Spearman correlation analysis showed that plasma and urine S1P levels and 24 h urinary protein excretion were positively correlated (*P* < 0.05).

**Figure 2:**
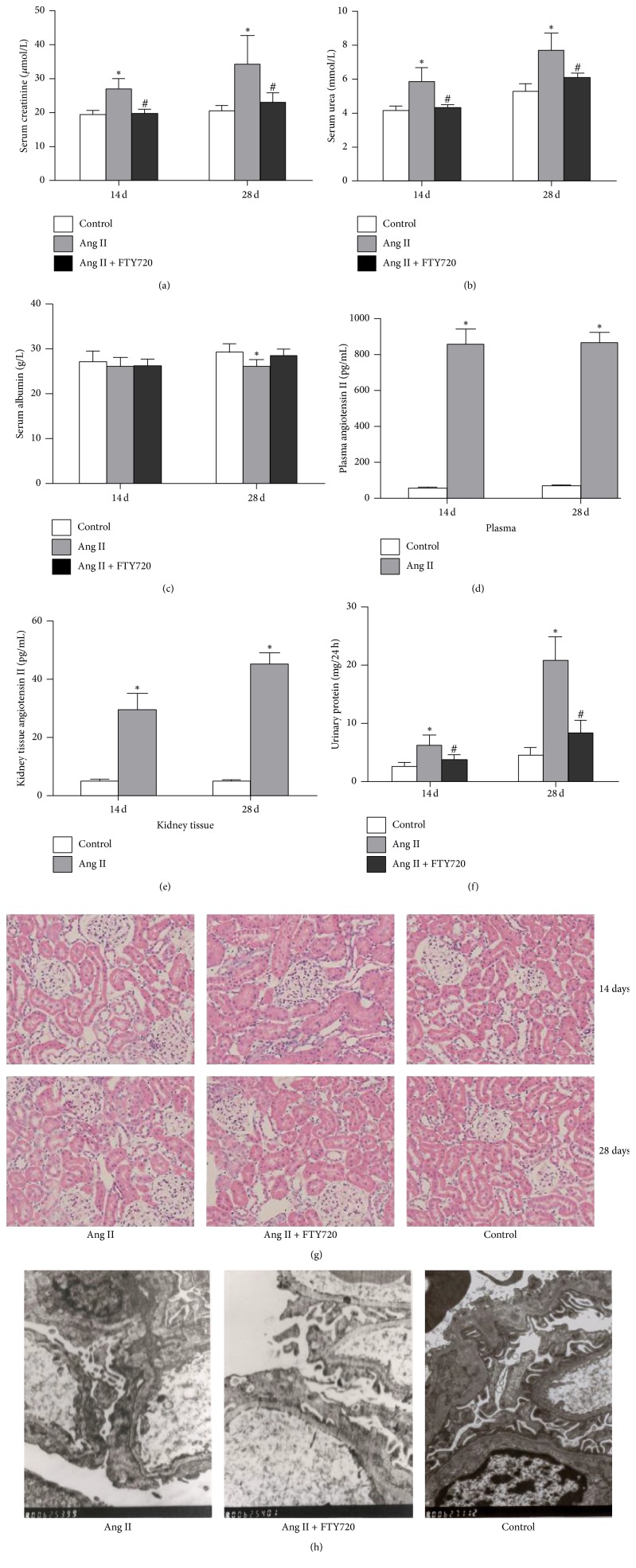
FTY720 could protect Ang II-induced kidney tissue injury in rats. The rats were subjected to subcutaneously continuous Ang II-infusion (400 ng/kg·min) for 14 or 28 days and were sacrificed. Rats given FTY720 (0.5 g/kg·d gavage) for 14 or 28 days also received Ang II-infusion. After the mice were sacrificed, the kidneys were fixed in paraformaldehyde for HE staining. The other parts of renal cortex were fixed in glutaraldehyde and podocytes were analyzed using transmission electron microscope. Kidney homogenate was used for RIA analysis. The plasma and serum were separated for biochemical analysis. Data represented is means ± SD of six independent experiments performed in triplicate. (*n* = 6) ^*∗*^*P* < 0.05, versus normal saline infusion control rats; ^#^*P* < 0.05, versus Ang II-infusion rats. (a) Serum creatinine levels in Ang II and FTY720-treated rats. (b) Serum urea nitrogen levels in Ang II and FTY720-treated rats. (c) Serum albumin levels in rats treated with Ang II and FTY720 for 14 and 28 days. (d) Concentration of Ang II in plasma from rats treated with Ang II and FTY720 for 14 and 28 days. (e) Concentration of Ang II in renal tissue homogenate from rats treated with Ang II and FTY720 for 14 and 28 days. (f) Twenty-four-hour urinary protein excretion levels in rats treated with Ang II and FTY720 for 14 and 28 days. (g) Light microscopy evaluation of rat kidney pathological changes with hematoxylin and eosin (H&E) staining (magnification, ×200). (h) Transmission electron microscopy evaluation of micrographs of podocyte ultrastructure in rats (magnification, ×8000).

Podocyte is a major component of the glomerular filtration barrier. Podocyte foot processes effacement is a characteristic hallmark of podocyte damage and associated with the onset of proteinuria [47]. Electron microscopic was used to analyze ultrastructural alterations of podocytes in Ang II-infusion and FTY720 intervention rats. Partial foot processes of podocytes are retracted and effaced in Ang II-infusion rats, resulting in displacement and disruption of the specialized slit diaphragms that span the filtration slits between foot processes. As shown in Figure 2(h), diffuse foot process fusion in podocytes and the basement membrane and diffuse uneven thickness in Ang II-infusion rats were observed under electron microscopy. Foot process fusion was often associated with detachment from the underlying glomerular basement membrane. Severe podocyte detachment from areas of denuded glomerular basement results in defects of glomerular filtration barrier, leading to development of severe proteinuria [48]. FTY720 intervention ameliorated these changes.

### 3.2. FTY720 Alleviated Renal Injury in Ang II-Infusion Rat

Our previous studies have found that systemic infusion of Ang II-infusion into normal rats caused renal pathological changes including glomerular mesangial cell proliferation, extracellular matrix deposition, tubular atrophy, dilation, urinary cast formation, inflammatory cell and inflammatory factors infiltration in tubulointerstitial lesion, and interstitial fibrosis [49, 50]. It is well known that Ang II results in the progression of glomerular injury via its hemodynamic or nonhemodynamic effects [51]. Here, a rat model of Ang II-infusion was established to evaluate the role of Ang II in glomerular, podocyte, and tubulointerstitial lesion.

As shown in Figure 2, serum creatinine, blood urea nitrogen, and 24-hour urinary protein excretion in 14 days or 28 days of Ang II-infused rats were higher than those in normal saline-infused control rats and they were significantly resorted by FTY720 pretreatment (*P* < 0.05) (Figures 2(a) and 2(b)). However, after Ang II-infusion for 14 days, the serum albumin concentration in rats showed no significant difference compared to the normal saline infusion control group (*P* > 0.05). However, after Ang II-infusion for 28 days, serum albumin concentration was significantly lower than the control group (^*∗*^*P* < 0.05) (Figure 2(c)). Ang II concentrations in both plasma and renal tissues detected by RIA in Ang II-infused rats were significantly increased versus control group (*P* < 0.05) (Figures 2(d) and 2(e)), thus indicating a successful Ang II-infusion rat model. 24-hour urinary protein excretion in Ang II-infused rats after 14 days or 28 days was higher than that in normal saline infusion control rats and was significantly blocked by pretreatment of FTY720, an S1PR agonist (*P* < 0.05) (Figure 2(f)). These results suggested that FTY720 blocked S1P-evoked physiological effects. After 28 days of Ang II-infusion, rat kidney histopathological changes were observed by HE staining. Compared with the normal saline infusion rats, light microscopy displayed significant mesangial and interstitial expansion, proximal tubular epithelial swelling, inflammatory cell infiltration in tubulointerstitial lesion in rats receiving 28 days of Ang II-infusion (Figure 2(g)). Ang II-infusion in rats on day 14 did not cause obvious renal histological lesions. Light microscopy only revealed mild mesangial and tubulointerstitial accumulation of matrix (Figure 2(g)). Thus, we demonstrated that 28 days of Ang II-infusion induced more obvious kidney damage in rats. FTY720 oral administration for 28 days dramatically reduced mesangial and tubulointerstitial accumulation of matrix, proximal tubular epithelial swelling, and inflammatory cell infiltration in tubulointerstitial lesion under light microscopy in Ang II-infusion rats (Figure 2(g)). Thus, we conclude that FTY720 can reduce renal histological lesions induced by Ang II-infusion in rats.

### 3.3. FTY720 Decreased S1P Synthesis in Ang II-Infusion Rats via Downregulation of Inflammatory Cytokines

**Figure 3:**
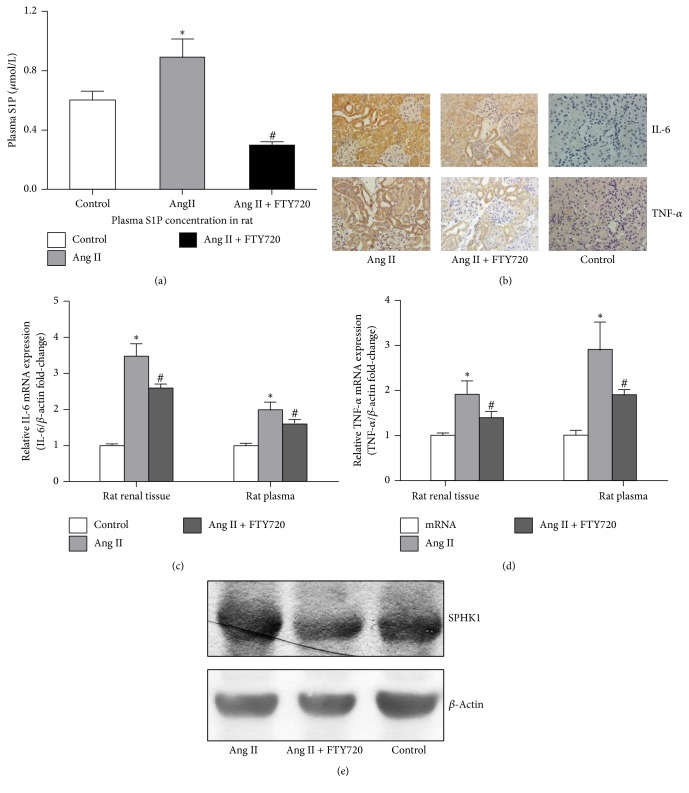
FTY720 decreased S1P synthesis in Ang II-infusion rats via downregulation of inflammatory cytokines. Subcutaneous continuous Ang II-infusion at 400 ng/kg·min concentration in rats for day 28: we sacrificed rats at day 28. FTY720 (0.5 g/kg·d) was orally administered to rats for 28 days and at the same time rats also received Ang II-infusion. When the mice were sacrificed, the kidney was fixed in paraformaldehyde; immunohistochemical analysis of TNF-*α* and IL-6 protein expression in kidney sections. Rat blood samples were collected and plasma was separated for analyzing S1P concentration by ELISA and SPHK1 protein expression level by western blot. Part of the rat kidney tissue homogenate was used to detect IL-6 and TNF-*α* mRNA expression level. Data represented is the means ± SD of 6 independent experiments performed in triplicate. (*n* = 6) ^*∗*^*P* < 0.05, versus normal saline infusion control rats; ^#^*P* < 0.05, versus Ang II-infusion rats. (a) S1P concentration in rat plasma were analyzed by ELISA. (b) TNF-*α* and IL-6 were detected by immunohistochemical in rat kidney. (c) IL-6 mRNA expression level was detected by RT-PCR in rat kidney tissue homogenate. (d) TNF-*α* mRNA expression level was detected by RT-PCR in rat kidney tissue homogenate. (e) SPHK1 protein expression level in rat plasma was detected by western blot. *β*-Actin was used to ensure an equal amount of protein was loaded in each lane. Data represented is the means ± SD of six independent experiments performed in triplicate.

After 28 days of Ang II-infusion, the animals were sacrificed. S1P concentrations in plasma and urine were measured using ELISA, but S1P concentrations in urine were not detectable. Plasma S1P levels in Ang II-infusion rats were much higher than those in control group. However, FTY720 intervention significantly decreased the concentrations of plasma S1P in Ang II-infusion rats (*P* < 0.05) (Figure 3(a)). Next, TNF-*α* and IL-6 expression in rat kidney tissues were detected by using an immunohistochemical technique. We demonstrated that TNF-*α* and IL-6 were mainly expressed in renal tubule after Ang II-infusion, and a very low level of IL-6 and TNF-*α* was expressed in glomeruli. Compared to the normal saline infusion rats, TNF-*α* and IL-6 expressions were elevated in Ang II-infusion rats. However, FTY720 administration inhibited TNF-*α* and IL-6 expression in the renal tissues from Ang II-infusion rats (Figure 3(b)), suggesting that FTY720 intervention can alleviate Ang II-infusion-induced rat renal tissue inflammation. The IL-6 and TNF-*α* mRNA levels in both renal tissue and rat plasma were also detected by real-time PCR. Ang II-infusion elevated the levels of IL-6 and TNF-*α* mRNA expression, which were inhibited by FTY720 intervention (Figures 3(c) and 3(d)).

Next, we investigated the mechanism underlying FTY720-induced decrease in S1P concentration. Sphingosine kinases (SPHK), including SPHK1 and SPHK2, are a conserved lipid kinase family that catalyzes S1P formation. SPHK1 is found in the cytosol of eukaryotic cells and migrates to the plasma membrane upon activation, whereas SPHK2 is localized to the nucleus. Here, SPHK1 protein expression was measured by western analysis. As shown in Figure 3(e), FTY720 intervention significantly inhibited Ang II-induced increase in rat plasma SPHK1 protein expression.

### 3.4. FTY720 Can Alleviate Exogenous S1P-Induced Podocyte Damage

**Figure 4:**
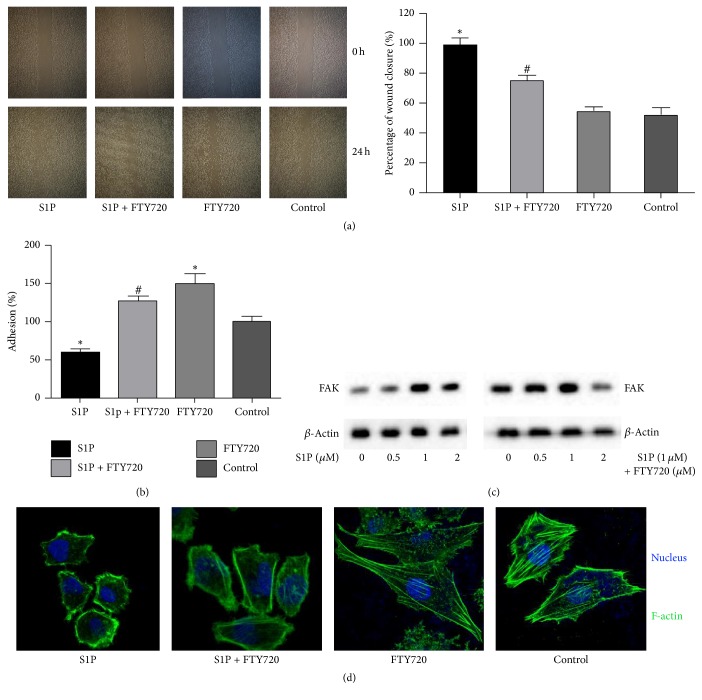
FTY720 could alleviate exogenous S1P-induced podocyte damage. Immortalized murine podocytes were cultured. Using 5 *μ*M FTY720 pretreated for 30 min, followed by S1P treatment (2 *μ*M) for 24 h. Using different doses of S1P within 0~2 *μ*M S1P treated podocyte for 24 h. Or using 5 *μ*M FTY720 treated for 24 h. Podocyte migration was measured by using wound-healing assay. Podocyte migration was measured by using adhesion assay and measured by MTT assay for adhesion cells in plate. FAK protein expression level in rat plasma was detected by western blot. *β*-Actin was used to ensure an equal amount of protein was loaded in each lane. Data represented is the means ± SD of six independent experiments performed in triplicate. Immunofluorescence was used to observe F-actin change. Three independent experiments were performed. Results are presented as mean cell migration velocity ± SD of the independent experiments, ^*∗*^*P* < 0.05, versus control podocyte; ^#^*P* < 0.05, versus S1P treated podocyte. (a) The migration ability of podocyte cells was measured using wound-healing assay. The percentage of wound closure was significantly reduced in response to treatment with either S1P or FTY720. Podocyte was treated with S1P which showed a higher cell migration velocity than FTY720-treated or control cells. Podocyte pretreated with FT720 and then treated with S1P showed a slower migration velocity than S1P treated podocyte. (b) The adhesion ability of podocyte cells was measured using adhesion assay. (c) FAK protein expression level in rat plasma was detected by western blot. (d) Representative confocal microscopy images from 3 separate experiments of podocyte cells were treated with S1P or FTY720 for 24 h. F-actin (stained with phalloidin): green; and nuclei: blue. Original magnification ×1,000.

To further substantiate the protective effects of FTY720 against S1P-induced damage, we pretreated cultured immortalized murine podocytes with 5 *μ*M FTY720 for 30 min, followed by S1P treatment (2 *μ*M) for 24 h. Podocyte migration was measured by using wound-healing assay. As shown in Figure 4(a), S1P accelerated podocyte migration, while FTY720 could inhibit S1P-induced podocyte migration. In addition, FTY720 significantly blocked S1P-induced inhibition of podocyte adhesion (*P* < 0.05) (Figure 4(b)). Focal adhesion kinase (FAK) is a nonreceptor tyrosine kinase that plays a critical role in cell motility [52]. Glomerular injury leads to activation of podocyte FAK [53]; thus FAK can be used as a podocyte injury marker. Here, we demonstrated that S1P increased FAK protein expression in a concentration-dependent manner. However, pretreatment with 5 *μ*M FTY720 could inhibit S1P-induced FAK protein expression (*P* < 0.05) (Figure 4(c)).

Next, we observed F-actin in podocyte. The expression of F-actin in the cytoplasm in S1P-treated group was lower than that in the control group. Moreover, in the normal podocyte stress fibers gathered by F-actin were arranged predominantly in one direction. After S1P treatment, some irregular actin filaments were observed with no stress fibers in the cell periphery. Importantly, FTY720 rescued S1P-induced cytoskeleton destruction (Figure 4(d)).

## 4. Discussion

The present study demonstrates a novel finding that FTY720 has protective effects against chronic kidney disease renal damage in an Ang II-infusion rat model. The concentration of S1P in serum and urine was positively correlated with chronic kidney disease renal injury. Moreover, FTY720 decreased S1P synthesis in Ang II-infusion rats via downregulation of inflammatory cytokines, including TNF-*α* and IL-6, and controlled glomerular permeability and podocyte function. FTY720 alleviated renal injury in Ang II-infusion rat. In addition, we observed that FTY720 can relieve exogenous S1P-induced podocyte damage. Angiotensin II (Ang II), the main effector peptide of the renin-angiotensin system (RAS), plays a central role in the pathophysiology of renal diseases. Apart from contribution to the progression of glomerular injury through its hemodynamic and/or nonhemodynamic effects, Ang II is considered as a cytokine with an active role in renal pathology. Since progressive kidney disease caused by Ang II arises from aberrations of the glomerulus and the tubulointerstitium, the wide ranging effects of S1PR agonists may be more efficient than current therapies.

Multiple receptor system empowers S1P to have pleiotropic actions and modulate a number of imperative cellular functions [54, 55]. The physiological functions of S1P have been studied extensively in various tissues. Nonetheless, the particular pathophysiology role of S1P in kidney is not clear. Kidney expresses S1PRs [25, 56], which play a significant role in sustaining endothelial cell integrity [57, 58] and in transferring lymphocytes [59]. S1P and SPHK1 are associated with the actions of TNF-*α*, a cytokine critical for inflammation and autoimmune disorders, for example, inflammatory bowel disease, rheumatoid arthritis, and asthma. TNF-*α* stimulates ERK1/2-mediated phosphorylation and translocation of SPHK1 to the plasma membrane, catalyzing the formation of S1P [60]. TRAF2, an important signaling intermediate in TNF-*α* pathway, activates NF-kB by binding directly to SPHK1 [61].

Specifically, S1P regulates vascular barrier integrity in inflammatory conditions and S1P1 fundamentally protects the vasculature from leak with countering effects of S1P2/3. Furthermore, S1P signaling controls the trafficking of several types of immune cells, such as lymphocytes, monocyte, macrophages, and neutrophils [62], influencing functions of these cells, for example, formation of inflammatory mediators. Therefore, therapeutic intervention targeting S1P pathway has advantageous effects. It would be important to develop tissue-/receptor subtype-specific interventions of S1P signaling.

TNF-*α* is secreted by macrophages and functions as a crucial inflammatory mediator regulating inflammation responses and immune cell activity [63]. TNF-*α* regulates cell growth by activating NF-kB, a proinflammatory transcription factor [64–66]. TNF-*α*/NF-kB are promising targets for developing novel chronic kidney disease drugs. It is intriguing to speculate that some drugs with inhibitory effect on TNF-*α* activation show imperative benefits for kidney damage. In the present study, we demonstrate that TNF-*α*, IL-6 expression, and S1P levels were elevated in Ang II-infusion rats. However, FTY720 administration inhibited TNF-*α*, IL-6 expression, and S1P content in the renal tissues from Ang II-infusion rats, suggesting that FTY720 intervention can alleviate Ang II-infusion-induced rat renal tissue inflammation. Our results illuminate that the TNF-*α*/S1P cascade-mediated kidney inflammatory response induced by Ang II can be repressed by FTY720. The kidney-protective effects of FTY720 might be associated, at least partially, with its ability to inhibit TNF-*α* and S1P activity.

In conclusion, this study deepens our understanding of the role of S1P in Ang II-induced kidney damage and reveals that administration of S1P1R agonists decreases renal dysfunction. The protective effect of S1P1R agonist on kidney tissue is mediated by a directly beneficial effect on kidney-derived cells, namely, podocytes. We conclude that S1P1R activation might represent a novel therapeutic approach for the early-stage chronic kidney disease.

## Figures and Tables

**Table 1:** Renal function and albumin Ang II-infusion rat and FTY720 intervention rat.

Index	Healthy volunteers	IgA nephropathy patients	*t*	*P*
Number	10	40	—	—
Sex (male/female)	6/4	18/22	—	0.60
Age (year)	24.8 ± 1.30	36.80 ± 13.77	17.03	0.07
Serum total protein (g/L)	75.9 ± 3.18	61.99 ± 7.91	1.89	<0.001
Serum albumin (g/L)	47.04 ± 2.23	34.93 ± 4.44	5.53	<0.001
Serum urea nitrogen (mmol/L)	3.80 ± 1.11	5.06 ± 1.20	1.06	0.15
Serum creatinine (*μ*mol/L)	48.80 ± 5.11	89.06 ± 63.20	1.98	0.18
24 h urinary protein excretion (g/24 h)	0.03 ± 0.01	2.43 ± 1.87	10.51	0.01
Cholesterol (mmol/L)	3.31 ± 0.14	4.53 ± 0.91	6.16	<0.001
Triglycerides (mmol/L)	1.28 ± 0.19	1.58 ± 0.64	7.16	<0.001
Hemoglobin (g/L)	126.20 ± 9.06	124.00 ± 20.60	4.37	0.32
Red blood cell (×10^12^/L)	4.12 ± 0.89	3.71 ± 1.23	3.54	0.89
